# Rotational assessment of distal femur and its relevance in total knee arthroplasty: analysis by magnetic resonance imaging*

**DOI:** 10.1590/0100-3984.2014.0037

**Published:** 2015

**Authors:** Fabricio Bolpato Loures, Sebastião Furtado Neto, Robson de Lima Pinto, André Kinder, Pedro José Labronici, Rogério Franco de Araújo Góes, Edson Marchiori

**Affiliations:** 1 Master, Knee Surgeon, Hospital Santa Teresa, Petrópolis, RJ, Brazil.; 2 Members of Sociedade Brasileira de Ortopedia e Traumatologia, MDs, Residents of Knee Surgery at Hospital Santa Teresa, Petrópolis, RJ, Brazil.; 3 Master, MD, Radiologist at Clínica Multimagem, Petrópolis, RJ, Brazil.; 4 PhD, MD, Orthopedist, Hospital Santa Teresa, Petrópolis, RJ, Brazil.; 5 Member of Sociedade Brasileira de Cirurgia do Joelho, Clinical Chief and Physician in Charge, Knee Group, Service of Orthopedics and Traumatology of Professor Dr. Donato D’Ângelo, Hospital Santa Teresa, Petrópolis, RJ, Brazil.; 6 PhD, Full Professor Emeritus, Universidade Federal Fluminense (UFF), Niterói, RJ, Associate Professor, Universidade Federal do Rio de Janeiro (UFRJ), Rio de Janeiro, RJ, Brazil.

**Keywords:** Knee, Prosthesis, Image, Magnetic resonance imaging, Alignment

## Abstract

**Objective:**

To define the distal femur rotation pattern in a Brazilian population, correlating such pattern with the one suggested by the arthroplasty instruments, and analyzing the variability of each anatomic parameter.

**Materials and Methods:**

A series of 101 magnetic resonance imaging studies were evaluated in the period between April and June 2012. The epidemiological data collection was performed with the aid of the institution’s computed imaging system, and the sample included 52 male and 49 female patients. The measurements were made in the axial plane, with subsequent correlation and triangulation with the other plans. The posterior condylar line was used as a reference for angle measurements. Subsequently, the anatomical and surgical transepicondylar axes and the anteroposterior trochlear line were specified. The angles between the reference line and the studied lines were calculated with the aid of the institution’s software.

**Results:**

The mean angle between the anatomical transepicondylar axis and the posterior condylar line was found to be 6.89°, ranging from 0.25° to 12°. For the surgical transepicondylar axis, the mean value was 2.89°, ranging from –2.23° (internal rotation) to 7.86°, and for the axis perpendicular to the anteroposterior trochlear line, the mean value was 4.77°, ranging from –2.09° to 12.2°.

**Conclusion:**

The anatomical transepicondylar angle showed mean values corresponding to the measurement observed in the Caucasian population. The utilized instruments are appropriate, but no anatomical parameter proved to be steady enough to be used in isolation.

## Abstract (pt)

**Objetivo:**

Definir o padrão de rotação do fêmur distal em população brasileira, correlacionar esse padrão com o sugerido pelos instrumentais de artroplastia e analisar a variabilidade de cada parâmetro anatômico.

**Materiais e Métodos:**

Foram avaliados 101 exames de ressonância magnética no período compreendido entre abril e junho de 2012. A coleta dos dados epidemiológicos foi feita pelo sistema informatizado da instituição de imagem, sendo 52 pacientes masculinos e 49 femininos. As mensurações foram feitas no plano axial, correlacionando e triangulando com os outros planos. Utilizamos como referência para as medidas angulares a linha condilar posterior. Na sequência, especificamos o eixo transepicondilar anatômico, cirúrgico e a linha troclear anteroposterior. As angulações entre a linha de referência e as linhas estudadas foram calculadas pelo *software* da instituição.

**Resultados:**

Foi encontrada uma média de 6,89° na aferição do eixo transepicondilar anatômico em relação à linha condilar posterior, variando de 0,25° a 12°. O eixo transepicondilar cirúrgico apresentou média de 2,89°, variando de –2,23° (rotação interna) a 7,86°. O eixo perpendicular à linha troclear anteroposterior apresentou média de 4,77°, variando de –2,09° a 12,2°.

**Conclusão:**

O ângulo transepicondilar cirúrgico apresentou valores médios correspondentes aos da população caucasiana. Os instrumentais estão adequados, porém nenhum parâmetro anatômico se mostrou constante o suficiente para ser usado de forma isolada.

## INTRODUCTION

The success of total knee arthroplasty depends on several factors, among them the implant positioning in the axial plane. Failure in implant positioning may result in disproportionate tension on the ligaments, causing complications such as development of pain, spasticity, instability or early loosening of the implant^(1-10)^. Several studies have demonstrated deleterious consequences from the positioning of the femoral component in internal rotation. Recently, some studies also demonstrated severe consequences in cases where the implant was positioned in excessive external rotation^(11-16)^.

The femoral section is made in external rotation to compensate for the tibial section perpendicular to the anatomical axis, since the tibial plateau originally presents with 3º varus deformity, also to place the implant parallel to the knee rotation axis and to improve the femoropatellar joint relationship. Thus, the correct rotation facilitates the ligament balancing, aiding in the balance of the extension and flexion gaps. There are several anatomical parameters to determine the correct rotational alignment, such as the Whiteside's anteroposterior trochlear line, the anatomical transepicondylar axis, the surgical transepicondylar axis, the posterior condylar line, and the anterior tangential line of the femur. However, taking their variability into consideration, none of those parameters should be utilized in isolation^(1-10)^.

Most total knee arthroplasty instruments utilize the posterior condylar line of the femur to guide the implant positioning with three-degree external rotation in the axial plane. Such a reference has shown to be appropriate in cases of neutral or varus knee alignment; but in cases of valgus knee deformity this is not an ideal reference^(1,10-13)^.

There is a recent questioning regarding the use of anatomical parameters as a reference for implants positioning, without considering the patient's own characteristics such as age, gender, height and race^(13,14)^.

The primary objective of the present study was to define the distal femur rotation pattern in a Brazilian population. The secondary objectives included correlating such a pattern with the one offered by the arthroplasty instruments and analyzing the variability of each anatomical parameter.

## MATERIALS AND METHODS

The authors evaluated 101 magnetic resonance imaging studies performed in the imaging clinic in the period from April to June/2012. The measurements were performed by two orthopedists, both titular members of Sociedade Brasileira de Ortopedia e Traumatologia and the second one, member of Sociedade Brasileira de Cirurgia do Joelho. The procedures were supervised by a radiologist, titular member of Colégio Brasileiro de Radiologia e Diagnóstico por Imagem.

The epidemiological data collection was performed with the aid of the institution's computed system, and the study sample included 52 male and 49 female patients.

The scans were performed in a 1.5 T apparatus (Magnetom Essenza; Siemens, Germany). The patients were examined in the supine position with the knee relaxed either in full extension or minimal flexion (< 15º), for more comfort. The following sequences were acquired: sagittal, proton density-weighted with fat suppression (repetition time (TR): 2800 ms; echo time (TE): 35 ms; slice thickness: 4 mm; field of view (FOV): 160/160 mm; matrix: 230/320); sagittal, T1-weighted (TR: 540 ms; TE: 13 ms, slice thickness: 4 mm; FOV: 160/160 mm; matrix: 230/384); coronal, proton density-weighted with fat suppression (TR: 2040 ms; TE: 32 ms; slice thickness: 4 mm; FOV: 160/160 mm; matrix: 224/320); and axial, proton density-weighted with fat suppression (TR: 3.140 ms; TE: 35 ms; slice thickness: 4 mm; FOV: 160/160 mm; matrix: 192/320).

**Figure 1:**
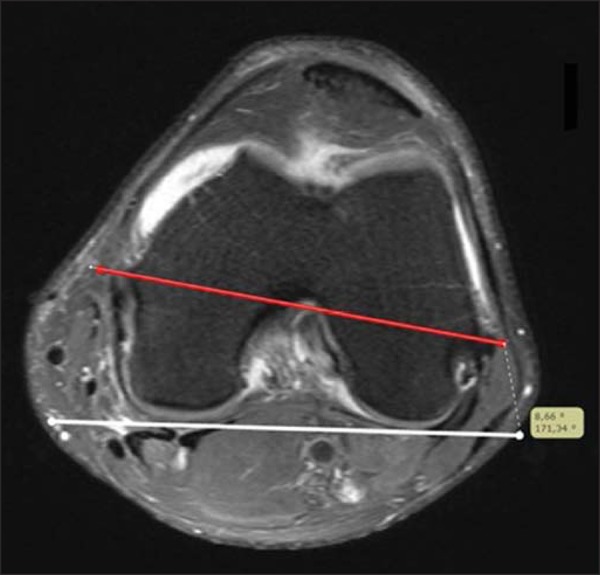
White line: posterior condylar line. Red line: anatomical transepicondylar axis.

**Figure 2:**
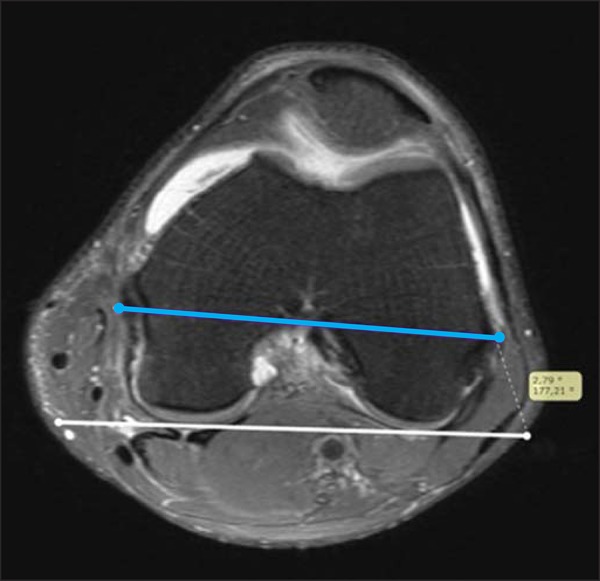
White line: posterior condylar line. Blue line: surgical transepicondylar axis.

**Figure 3:**
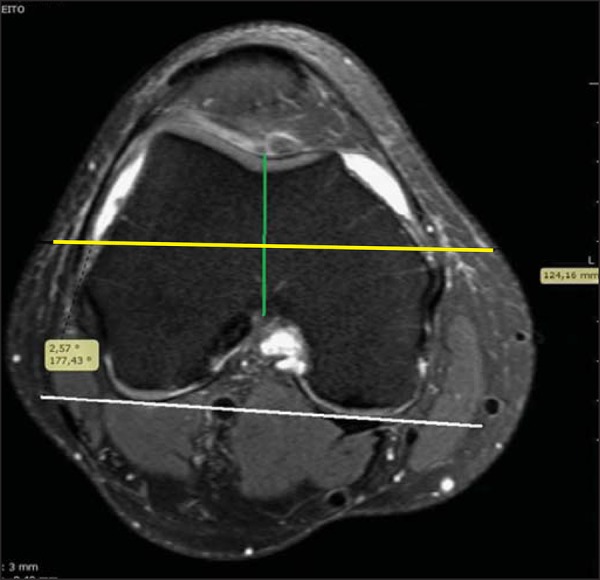
White line: posterior condylar line. Green line: Whiteside’s line. Yellow line: perpendicular to the Whiteside’s line.

The measurements were made in the axial plane, with subsequent correlation and triangulation with the other planes. The posterior condylar line drawn tangentially to the posterior aspects of the femoral condyles was used as a reference for angle measurements. Subsequently, the anatomical and transepicondylar axis was specified using the bone prominences of the medial and lateral epicondyles as reference (Figure 1). The surgical transepicondylar axis was drawn, utilizing the center of the medial epicondylar groove and the lateral epicondyle as anatomical references (Figure 2). Finally, the Whiteside's anteroposterior trochlear line was drawn, using as anatomical reference the trochlear notch and the center of the femoral intercondyle. The angles between the perpendicular line and the reference line and the posterior condylar line were measured (Figure 3).

The angles between the posterior condylar line (reference line) and the studied lines were calculated with the aid of the imaging processing software OsiriX^®^.

The categorical and numerical data were presented as descriptive tables.

The inferential analysis was composed with the Student's *t* test for independent samples in the comparison of the clinical data and angles in female and male patients. The Pearson's correlation coefficient was utilized to evaluate the degree of association between angles measurements with the clinical variables. The one-way ANOVA was applied to compare the numerical clinical variables between the three ranges of the surgical angle, and the χ^2^ test to compare categorical data adopting 5% as significance level. The statistical analysis was undertaken with the aid of the statistical software SAS^®^ System, version 6.11.

## RESULTS

The present study sample included 101 patients divided into two groups according to sex, as follows: 52 (51.5%) men and 49 (48.5%) women. The male and female groups showed to be equivalent in terms of age and body mass index. As regards weight and height, the male group presented with significantly higher values (Table 1).

**Table 1:** General descriptive table of clinical variables.

Variable	Mean	SD	Median	Q1 - Q3	Minimum	Maximum
Age (years)	39.9	15.3	40.0	29 - 53	13	84
Weight (kg)	75.6	13.8	74.0	67.5 - 86	36	108
Height (m)	1.68	0.09	1.70	1.60 - 1.75	1.50	1.90
BMI (kg/m^2^)	26.6	4.1	26.9	23.6 - 29.3	15.2	38.6

SD, standard deviation; Q1, 1st quartile; Q3, 3rd quartile, BMI, body mass index.

The mean value of the angle between the anatomical transepicondylar axis and the posterior condylar line was 6.89º, ranging from 0.25º to 12º. The mean angle between the surgical transepicondylar axis and the reference line was 2.89º, ranging from -2.23º (internal rotation) to 7.86º. The axis perpendicular to the Whitesides's anterior trochlear line presented a mean angle of 4.77º, ranging from -2.09º to 12.2º.

The angle between the anatomical transepicondylar axis and the posterior condylar line demonstrated the lower variability, with 30.8%, followed by the Whiteside's anterior trochlear line, with 58.7%, and, finally, the angle of the surgical transepicondylar axis, with variability of 69%. The anatomical transepicondylar angle demonstrated to be the most constant (Table 2).

**Table 2:** Descriptive table of the three angle measurements in the 101-patient sample.

Angle (degrees)	Mean	SD	Median	Q1 - Q3	Minimum	Maximum	CV (%)
Anatomical	6.89	2.12	7.04	5.58 - 8.36	0.25	12.0	30.8
Surgical	2.89	1.99	3.25	1.36 - 4.39	-2.23	7.86	69.0
Whiteside's line	4.77	2.80	4.80	2.82 - 6.92	-2.09	12.2	58.7

SD, standard deviation; Q1, 1st quartile; Q3, 3rd quartile; CV, coefficient of variation.

No significant difference was observed between male and female patients as regards measurements of the angles of the anatomical axis (*p* = 0.34), surgical axis (*p* = 0.47) and perpendicular to the Whiteside's anterior trochlear line (*p* = 0.090). However, there is a tendency toward a greater rotation in relation to the Whiteside's line in the male patients as compared with the female patients, with *p* = 0.090 (Table 3).

**Table 3:** Measurements of angles according to sex.

Angle (degrees)	Sex	Mean	SD	Median	Q1 - Q3	Minimum	Maximum	*p*-value*
Anatomical	Male	7.08	2.00	7.03	5.40 - 8.68	0.92	12.0	0.34
	Female	6.68	2.25	7.04	5.64 - 8.1	0.25	10.9	
Surgical	Male	3.03	2.02	3.41	1.43 - 4.56	-1.81	7.86	0.47
	Female	2.74	1.98	3.14	1.33 - 3.85	-2.23	6.32	
Whiteside's line	Male	5.23	2.86	5.0	3.13 - 7.07	0.72	12.2	0.09
	Female	4.28	2.68	4.2	2.69 - 6.69	-2.09	9.8	

SD, standard deviation; Q1, 1st quartile; Q3, 3rd quartile.

* Student *t* test for independent samples.

## DISCUSSION

With the enhanced development of the total knee arthroplasty instruments, the frequency of placement of femoral implants with inappropriate rotation has decreased. However, the rotation provided by the instrument may not be ideal, so the surgeon should know the anatomical parameters for a correct implant positioning^(1-10)^.

Despite radiographic images demonstrating a correct implant positioning, unsatisfactory clinical outcomes are observed in some patients. Because of such unfavorable and not so well understood situations, one has raised the possibility that constitutional alterations specific to a race or sex would be generating such outcomes. This should be justified by anatomical differences between populations^(13,14)^. In this context, some studies have demonstrated rotational differences between men and women, while others have demonstrated differences related to age^(1,2,6)^.

In the daily practice, one faces frequent structural differences between patients. The same concept could be applied as regards nationalities. With the knowledge about such constitutional alterations, one could develop more appropriate rotational parameters for a specific gender or population, reducing the frequency of poor clinical outcomes.

In the present study, the authors found the anatomical transepicondylar axis as the reference line with least variation among the patients (30.8%), and mean value of 6.89º. In a literature review, the authors found four studies from 1987 to 2007 approaching the relation between the posterior condylar line and the anatomical transepicondylar axis. Such studies reported a mean value of 5.52º, ranging from 3.5º to 6.8º. The present study sample showed a slightly superior result as compared with other studies in the literature^(1,4,6,8)^.

As regards the surgical transepicondylar axis, the authors have found three studies approaching its relation with the posterior condylar line, obtaining a mean value of 3.19º, ranging from 0.3º to 5.4º. The present study has found a mean value of 2.89º, which can be considered to be in agreement with other studies^(1,2,5)^. The interindividual variability was of 69%, thus being the most inconstant amongst the studied axes.

As regards the Whiteside's anterior condylar line, the authors have found two studies calculating the angle with that line perpendicular to the posterior condylar line. Such studies have found angles of 3.8º and 3.1º, obtaining a mean value of 3.45º as compared with 4.47º found in the present study, and, again, demonstrating a slightly superior value as compared with other studies^(11,12)^.

The epidemiological data have not demonstrated any statistically significant alteration as regards age and sex, although the male patients have presented with a tendency to have a line perpendicular to the Whiteside's line with greater angulation. Also, in the literature, the authors have not found any study with statistically significant differences in such an aspect justifying further investigation^(1,2,6)^.

A limitation of the present study was the lack of intraobserver analysis. The strength of the study was the use of magnetic resonance imaging, while most of the cited studies were based on computed tomography, that do not consider the cartilage of the femoral condyles to draw the reference line. This may have contributed to the slight increase in the rotation observed in the present study^(17-21)^. Taking into consideration the fact that, in the surgical procedure, the cartilage is utilized as a reference, it seems to be more appropriate to include such a structure in the rotational measurement. This influence can be confirmed by other studies that demonstrated a greater angulation in elder patients due to the decreased thickness of the posterior articular cartilage^(1,2)^.

## CONCLUSION

The surgical transepicondylar angle presented mean values corresponding to those observed in the Caucasian population. Thus, the total knee arthroplasty instruments developed for such a population, suggesting a femoral section of 3º external rotation can be utilized in Brazil without the need for rotational adjustment. But no measurement demonstrated to be constant sufficient to be used in isolation. The surgeon must be prepared for the discrepant cases, by knowing the different anatomical references to minimize the chance of rotational error and consequential deleterious outcomes.
